# New 12S metabarcoding primers for enhanced Neotropical freshwater fish biodiversity assessment

**DOI:** 10.1038/s41598-020-74902-3

**Published:** 2020-10-21

**Authors:** David T. Milan, Izabela S. Mendes, Júnio S. Damasceno, Daniel F. Teixeira, Naiara G. Sales, Daniel C. Carvalho

**Affiliations:** 1 Conservation Genetics Lab, Postgraduate Program in Vertebrate Biology, Pontifical Catholic University of Minas Gerais, PUC Minas, Belo Horizonte, Brazil; 2 Postgraduate Program in Genetics, Institute of Biological Sciences, Federal University of Minas Gerais, Belo Horizonte, Brazil; 3 Ecosystems and Environment Research Centre, School of Environment and Life Sciences, University of Salford, Salford, UK; 4 CESAM - Centre for Environmental and Marine Studies, Departamento de Biologia Animal, Faculdade de Ciências da Universidade de Lisboa, Lisbon, Portugal

**Keywords:** Population genetics, Genetics, Ecology

## Abstract

The megadiverse Neotropical fish fauna lacks a comprehensive and reliable DNA reference database, which hampers precise species identification and DNA based biodiversity assessment in the region. Here, we developed a mitochondrial 12S ribosomal DNA reference database for 67 fish species, representing 54 genera, 25 families, and six major Neotropical orders. We aimed to develop mini-barcode markers (i.e. amplicons with less than 200 bp) suitable for DNA metabarcoding by evaluating the taxonomic resolution of full-length and mini-barcodes and to determine a threshold value for fish species delimitation using 12S. Evaluation of the target amplicons demonstrated that both full-length library (565 bp) and mini-barcodes (193 bp) contain enough taxonomic resolution to differentiate all 67 fish species. For species delimitation, interspecific genetic distance threshold values of 0.4% and 0.55% were defined using full-length and mini-barcodes, respectively. A custom reference database and specific mini-barcode markers are important assets for ecoregion scale DNA based biodiversity assessments (such as environmental DNA) that can help with the complex task of conserving the megadiverse Neotropical ichthyofauna.

## Introduction

Assessing biodiversity in species-rich regions is fundamental for environmental conservation as anthropogenic activities are drastically increasing the rate of biodiversity loss and changing ecosystem functioning^1^. Freshwater ecosystems are currently considered a priority target for biodiversity conservation due to the reported massive decline (i.e. ~ 83% since 1970) in species richness^2^. Therefore, aquatic ecosystem assessment and biomonitoring programs are conducted to provide data on fish species conservation status and community changes^3^. However, these programs are mostly based on traditional assessment methods (e.g. netting, trawling) and depend extensively on capture or observation^4^, which may be inefficient or cause harmful impacts to the environment and the biological communities^5^. Hence, developing alternative tools to monitor biodiversity is pivotal to inform conservation and management strategies^6^.

A promising alternative to traditional aquatic ecosystem assessment and biomonitoring methods is a DNA-based approach, which can complement or even be more efficient than traditional methods^7,8^. Further, it is possible to obtain DNA mixtures from environmental samples (e.g. water and sediment) without first isolating target organisms (environmental DNA – eDNA). After extraction, these samples can be subjected to high-throughput sequencing (HTS) to identify the presence of multiple species, namely DNA metabarcoding^9,10^.

DNA metabarcoding is a powerful tool for biodiversity assessment that has been widely used for several purposes and different taxonomic groups, including identification and quantification of neotropical ichthyoplankton^11,12^, stomach‐content analysis of a ray species^13^, and identification of wasp species using and comparing the Sanger and HTS methods^14^. Furthermore, environmental sampling (eDNA) has been successfully used for molecular identification of several vertebrate groups in temperate regions^15–17^, monitoring of endangered species such as freshwater fish in Australia and turtles in the United States^18,19^, and improved detection over traditional assessment methods for monitoring the invasive American bullfrog in France^20^. Additionally, Reid et al. (2019) highlight the use of eDNA as one of the main conservation and management tools for dealing with the emerging threats for freshwater biodiversity. However, the potential of DNA metabarcoding to monitor vertebrate communities remains poorly explored in the Neotropical region, and few studies have been conducted to date^11,21–23^.

The relative lack of DNA-based monitoring in the Neotropics may be due to some constraints that hamper its full application, such as incomplete taxonomic assignment due to the lack of reference sequences^21,22^. Therefore, the construction of a curated and complete reference molecular database is vital for efficient application of DNA-based methods towards biodiversity assessment in megadiverse realms. In the absence of reference sequences, taxonomic assignment is hindered, restricting the analyses to the use of Molecular Operational Taxonomic Units (MOTUs) and often only allowing assignments up to the family level, limiting ecological conclusions. The need for short amplicon length, due to DNA degradation in environmental DNA samples^24^, and for avoiding amplification of non-target taxa (e.g. invertebrates) are other pitfalls for sound DNA-based ecological monitoring, especially in biodiverse environments such as the Neotropics^21^.

A large dataset built using the DNA barcoding marker sensu stricto (i.e. use of ~ 600 base pairs (bp) of the mitochondrial cytochrome oxidase subunit I—COI) combined with traditional morphological techniques has contributed to the improvement of reference databases and to a better assessment of the Neotropical megadiverse ichthyofauna^25–28^. However, usage of the COI gene for macro-organism DNA metabarcoding analyses has proven to be difficult due to non-target amplification of bacteria and small microeukaryotes, which is inherent to the use of COI in eDNA samples^29^.

The 12S and 16S ribosomal RNA genes (rRNA) have been widely used as alternative markers and have provided efficient results for molecular detection of several species through eDNA metabarcoding, including fishes^7,21,30,31^. For instance, Miya et al. (2015)^32^ developed a 12S set of universal PCR primers for eDNA metabarcoding (MiFish) by targeting a hypervariable region with 163–185 bp from whole mitogenomes of 880 fishes, mostly subtropical marine species. Another 12S primer set commonly used in metabarcoding studies, Teleo1, was designed to amplify a region shorter than 100 bp based on 117 standard Teleostei (bony fish) sequences of the European Molecular Biology Laboratory—European Nucleotide Archive database^7^. However, there is also a need to use human blocking primers to avoid cross amplification. These markers were successfully applied in eDNA studies of high-diversity environments within the Neotropical freshwater ichthyofauna^21,22^ but without any previous analysis of marker taxonomic resolution or species detection efficiency.

Therefore, before applying DNA metabarcoding in the Neotropics, the development and validation of molecular markers that can provide a reliable and robust taxonomic assignment is highly recommended. To this end, MacDonald & Sarre (2017)^33^ suggested a framework for the development and validation of taxon-specific primers for eDNA metabarcoding analyses in ecological studies. This framework includes the construction of a reference database and its phylogenetic evaluation, primer design, and the in silico and in vitro evaluation of primer specificity, sensitivity, and utility in the laboratory.

Here, we developed a reference database targeting the 12S rRNA, using an *in-silico* approach, designed three new mini-barcoding 12S primer sets based on our reference database, and evaluated their phylogenetic resolution. The taxonomic resolution of full-length and mini-barcodes for species delimitation were compared using Bayesian and distance-based methods. In addition, we determined the genetic distance threshold value for fish species delimitation using the targeted 12S region for both mini and full-length libraries. In vitro tests were also conducted to validate our new 12S mini-barcode marker. Our custom reference database and new primer sets may be an alternative to previously developed markers to target Neotropical freshwater biodiversity and assist in the complex task of monitoring and conserving such diverse ichthyofauna.

## Materials and methods

### Tissue samples collection

We used fin clips from 67 fish species collected prior to this study and stored in 100% ethanol at − 4 °C at the Conservation Genetics Lab—PUC Minas. The specimens are from the São Francisco River Basin (Brazil), and the tissue samples and vouchers were previously used to build a DNA reference Barcode database using 650 bp of the Cytochrome oxidase I gene (ICMBIO collection permit number: 37298-1) from which the barcode data indicated cryptic species that would result in greater number of molecular taxonomic units. We followed the taxonomic classification obtained by Carvalho et al. (2011)^28^ through morphological and DNA barcoding for all fish. Additional information regarding DNA extraction, amplification, and sequencing is provided in the Supplementary Material (page 1).

### Sequences analyses

DNA was extracted using a salting-out protocol adapted from Aljanabi & Martinez (1997)^34^. Polymerase Chain Reactions (PCR) of the 12S rRNA gene were performed in a PCR thermal cycler (Veriti, Life Biosystems) using 10.0 μl solution composed of 7.0 μl of ultrapure water (Promega), 1.0 μl of 10X buffer containing 2.5 mM MgCl_2_, 1.0 μl of template DNA, 0.3 μl of dNTP (10 mM) (Invitrogen), 0.25 μl of each primer (10 μM), and 0.2 μl of Taq DNA polymerase (5U/ μl) (PHT). In order to amplify a fragment of ca. 600 bp of the 12S region (namely full-length region), we used the V05F_898 (5′-AAACTCGTGCCAGCCACC-3′) and teleoR (5′-CTTCCGGTACACTTACCATG-3′) primer sets presented in Thomsen et al. (2016)^35^. The thermal cycle profile consisted of initial denaturation at 95 °C (2 min), then 35 cycles of denaturation at 95 °C (1 min), primers annealing at 57 °C (30 s) and extension at 72 °C (1 min), and final extension at 72 °C (7 min). The amplicons were visualized in agarose 1% electrophoresis before DNA sequencing.

All samples were sequenced bi-directionally. The DNA sequencing reaction was performed using a Big Dye Terminator v.3.1 (Applied Biosystems) commercial kit in a reaction with a 10.0 μl final volume that consisted of: 1 μl of PCR amplified product, 1 μl of primer (10uM), 1 μl of Pre-Mix solution (Big Dye Terminator), 1.5 μl of Buffer 5X, and 5.5 μl of ultrapure water. The DNA sequencing reaction was performed in a Veriti thermocycler (Life Biosystems) with the following conditions: denaturation at 96 °C (2 min), then 35 cycles of denaturation at 96 °C (30 s), annealing at 50 °C (15 s), and extension at 60 °C (4 min). The samples were precipitated with EDTA (125 mM) and ethanol (100%) and washed with 70% ethanol. The sequencing plates were dried at 95 °C for eight minutes. DNA sequences were obtained in an ABI 3500 Genetic Analyzer (Life Technologies) automatic DNA analyzer.

The 12S consensus sequences (contigs) were obtained using DNA Baser v.3.5.4 software and aligned using ClustalW^36^, after trimming ambiguous ends. MEGA v7.0 software^37^ was used to estimate all genetic distances (intraspecific, intrageneric, intrafamilial, and interspecific) using the Kimura two-parameter (K2P) nucleotide evolution model^38^ and to construct dendrograms using the Neighbor-joining (NJ) method^39^, with 10,000 bootstrap pseudoreplicates^40^, showing only well-supported clade values (> 80%).

### Design and screening for best annealing primer sites

Three mini-barcode primer sets (NeoFish_1, NeoFish_2, and NeoFish_3) were designed to anneal to highly conserved flanking regions targeting variable sequences based on the alignment of all 12S DNA sequences obtained, which included 132 sequences corresponding to 67 species (19 species with only one specimen), ranging from one to three specimens/species (mean of 1.97 sequences per species). We used PRIMER3 software, implemented in Geneious v.4.8.5 (Kearse et al. 2012^41^—https://www.geneious.com) to find the best primer sites based on the 12S reference database by applying default parameters but restricting an amplicon length to shorter than 250 bp. Primers were designed with a 20%–80% guanine-cytosine (GC) content and a melting temperature between 57 and 63 °C. The best primer set was chosen based on an in vitro test, and it was then used for further analyses.

Evaluation of the newly developed primer sets was performed using a sliding window analysis (SWAN)^42^ conducted in the SPIDER package^43^ in R (version 3.6.1)^44^, which possesses useful analyses for determining ideal regions for mini-barcode design^45^. The *slideAnalyses* function was used to generate windows of 70 bp, which were shifted along the length of the 12S alignment in 10 bp intervals to evaluate regions of: (1) high mean K2P distance; (2) few zero pairwise non-conspecific distances; (3) high proportion of clades shared between the Neighbor-joining tree from the 12S full-length barcode and the tree constructed using only data from selected windows; and (4) high sum of diagnostic nucleotides.

Using the Primer Map analysis we check for overlapping amplification target regions of our newly developed mini-barcode primer set with previously developed 12S markers (i.e. MiFishU^32^, Teleo1^7^ and V05F_898^35^). The complete 12S rRNA sequence from *Prochilodus costatus* mitogenome (952 bp—GenBank number NC_027690) was used as a template.

To compare the non-target organism amplifications between our mini-barcode primer set to previously developed 12S markers (i.e. MiFishU and Teleo1), we performed in silico PCR using Primer-BLAST^46^. For primer specificity stringency options, we allowed at least three mismatches to unintended targets, including at least three mismatches within the last five base pairs at the 3′ end, a maximum target size of 400 bp and an annealing temperature of 60 °C.

### Species delimitation analyses of the full-length and mini-barcode reference database

The MOTUs were obtained to assess the taxonomic resolution of the full-length library (565 bp) and 193 bp mini-barcode (618–851 bp of *P. costatus* 12S complete sequence) from the trimmed 12S full-length reference by applying four single-locus species delimitation analyses. Two of these analyses were conducted using the Bayesian methods of Generalized Mixed Yule-Coalescent (GMYC)^47^ and Bayesian implementation of Poisson Tree Process (bPTP)^48^. Two other analyses used genetic distance-based methods: Automatic Barcode Gap Discovery (ABGD)^49^ and species delimitation threshold defined by threshold optimization analysis in SPIDER package. Each analysis was conducted as described below.

For GMYC, an ultrametric tree was generated for each marker by the Bayesian Phylogenetic reconstruction in BEAST^50^ and used as the input file. The relaxed lognormal distribution and the Birth and Death process as tree priors were used as clock models. The GTR + G + I model was used as nucleotide evolution model for 12S full-length and mini barcodes, and the Markov Chain (MCMC) procedure was used with 50 × 106 and 150 × 106 generations for 12S mini and full barcodes, respectively, sampling one tree every 104 generations. Convergence was indicated by Tracer v1.6^51^ with estimated sample sizes (ESS) superior to 200. An appropriate number of trees (first 10%) from each run was discarded as burn-in and the MCMC samples were generated using the maximum clade credibility (MCC) topology in TreeAnnotator v1.4.7^52^ and visualized in FigTree v1.4.3. The annotated trees were submitted for GMYC analysis in R with the Splits package (Species Limits by Threshold Statistics; https://r-forge.r-project.org/projects/splits) and a single threshold strategy using default scaling parameters.

We used the bPTP model in the bPTP web server (https://species.h-its.org/ptp/) under default parameters to delimitate the MOTUs. bPTP does not require an ultrametric gene tree and uses, instead, a Newick tree as the input file with branch lengths representing the number of nucleotide substitutions. We used Newick trees generated in MEGA7 as input files, using a Neighbor-joining method and the TN93 + G evolution model, which was chosen as the best evolutionary model in MEGA.

ABGD was applied to automatically group species into partitions indicating the molecular taxonomic resolution of the 12S database. ABGD first uses a range of prior intraspecific divergences to divide the data into groups based on a statistically inferred barcode gap and then recursively applies the same procedure to the groups obtained in the first step. ABGD analysis was performed using a web interface (https://wwwabi.snv.jussieu.fr/public/abgd/) with a relative gap width value of X = 0.8, while the other parameter values employed defaults. Assignments for intraspecific divergence (P-distances) between 0.001 and 0.100 were recorded^49^.

Threshold optimization analysis (SPIDER package) was conducted using the *threshVal* and *threshID* functions. A genetic distance-based species delimitation analysis was estimated using threshold values determined by the *threshVal* function. This function shows the number of true positive, true negative, false negative, and false positive, rate of fish species identification, together with the cumulative error (i.e. the sum of false positives and false negatives) using a range of threshold values based on K2P genetic distances. These estimated interspecific genetic distance thresholds were applied as the best cut-off values to delimitate species, as there are no previous references delimiting cut-off values for the 12S marker, unlike the COI gene (the 2% standard threshold defined by Ward, 2009^53^). Then, we used the distance threshold defined by *threshVal* in the *threshID* function. The *threshID* function assigns four possible results for each sequence in the dataset: “correct”, “incorrect”, “ambiguous”, and “no id”, where “correct” means that all matches within the threshold of the query are the same species and “no ID” shows that no matches were found to any individual within the threshold. Specimens identified as “no ID” were put in individual MOTUs and “correct” ones were put alongside their peers.

In addition, two distance-based analyses were performed (also using SPIDER) to identify taxa with low taxonomic resolution with the mini-barcode: barcoding gap and nearest Neighbor. Singleton sequences (19) were excluded. Detailed information about these analyses is provided in Supplementary Material (page 2).

### In vitro tests: evaluation of primer efficiency

To evaluate the efficiency of our mini-barcodes primers in amplifying DNA extracted from fish tissue samples and environmental samples, we conducted two tests. The first in vitro test consisted of PCR amplification and sequencing of the 12S mini-barcode region using the three newly developed primer sets with 16 fish species (22 samples). These samples had been previously used to develop the 12S reference database and represent the six major neotropical orders. PCR conditions for this test consisted of initial denaturation at 95 °C for 1 min, then 35 cycles of denaturation at 95 °C for 30 s, primers annealing at 60 °C for 30 s and extension at 72 °C for 1 min, and final extension at 72 °C for 7 min. DNA sequencing was conducted the same way as in the reference database construction section. For the second test, a water sample collected in an 80-L aquarium containing multiple individuals of pearl cichlid (*Geophagus brasiliensis*) was used to conduct an eDNA experiment to evaluate the potential use of our newly developed marker to detect fish DNA extracted from the environment (detailed information is provided in Supplementary Material, page 6). Experimental procedures followed the principles established by the Brazilian College of Animal Experimentation (COBEA) and approved by the Ethics Committee of the Pontifícia Universidade Católica de Minas Gerais (CEU PUC Minas – permit number: 021/2017).

## Results

### Custom reference database construction of full-length 12S

We sequenced 132 specimens from 67 fish species representing 54 genera, 25 families, and six orders: Characiformes (60.5% of species), Siluriformes (26%), Cyprinodontiformes (4.5%), Perciformes (4.5%), Gymnotiformes (3%), and Synbranchiformes (1.5%), with an average of 1.97 individuals per species (Supplementary Table S1). The 12S contigs were 565 bp long after trimming the ambiguous ends and had a nucleotide composition of 31.81% adenine, 26.84% cytosine, 20.4% guanine, and 20.95% thymine.

**Table 1:** Primers designed for short amplifications of the 12S sequences obtained from the major Neotropical fish orders.

Primer set	Primer name	Amplicon Length (bp)	Primer sequence (5′–3′)
NeoFish_1	NeoFish_1F	177	GCCGTCGCAAGCTTACCCTGT
	NeoFish_1R	177	GTGTGCGCGTCTCAGAGCCT
NeoFish_2	NeoFish_2/3F	184	CGCCGTCGCAAGCTTACCCT
	NeoFish_2R	184	GCGGTGTGTGCGCGTCTCAG
NeoFish_3	NeoFish_2/3F	193	CGCCGTCGCAAGCTTACCCT
	NeoFish_3R	193	AGTGACGGGCGGTGTGTGC

### Design and screening for best annealing primer sites

**Figure 1:**
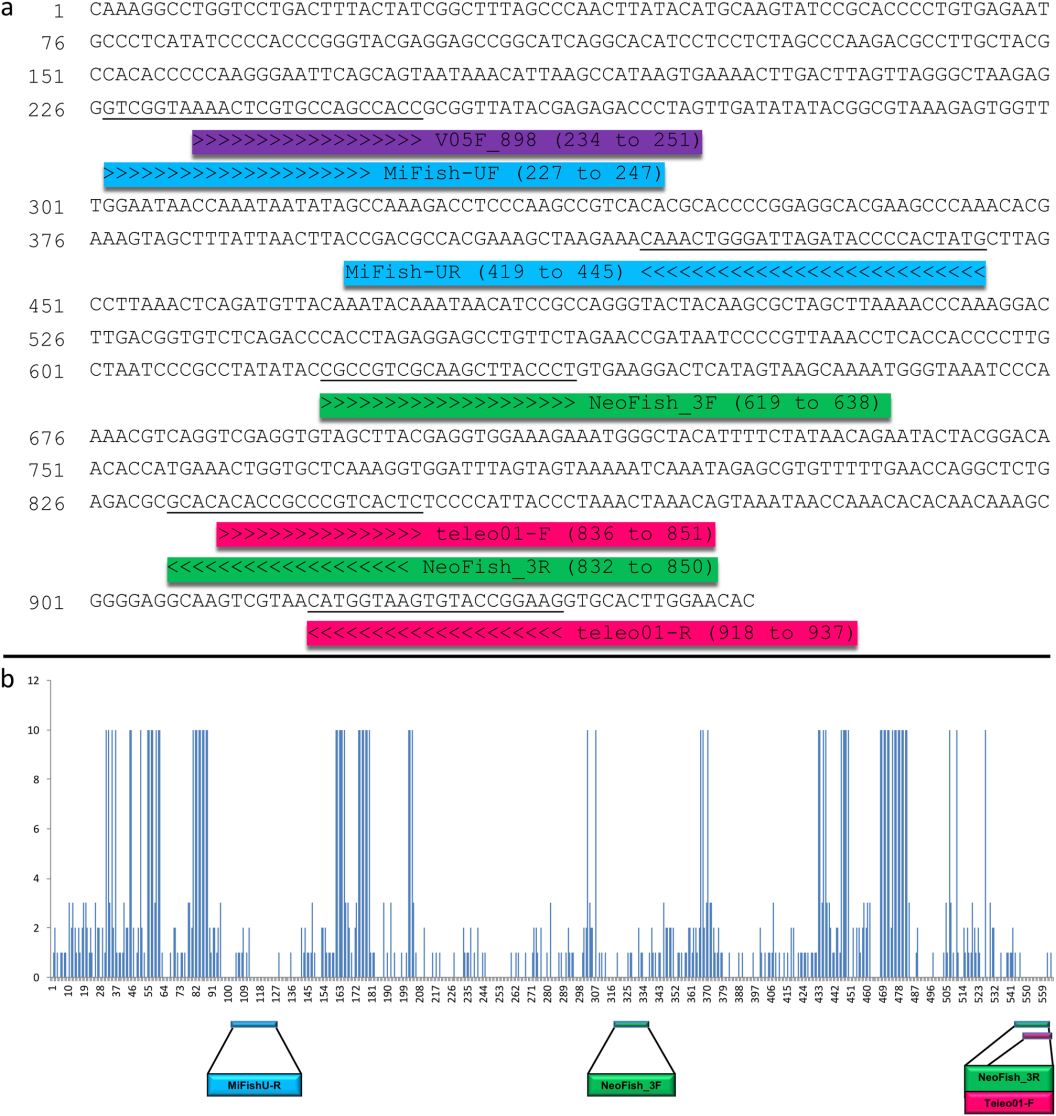
(**a**) DNA sequence alignment showing the annealing positions (< <  <  <  <  <  < <) for NeoFish_3 forward and reverse primers, V05F_898 (used for reference database construction), and for two others previously developed 12S markers (MiFishU and Teleo1). Base pairs highlighted in bold represent positions where the full-length database starts (317 bp) and ends (851 bp) (**b**) Mismatches and primer sites from the full 12S alignment (565 bp) considering 132 sequences of 67 fish species. Mismatches equal to 10 represent gap sites.

**Figure 2:**
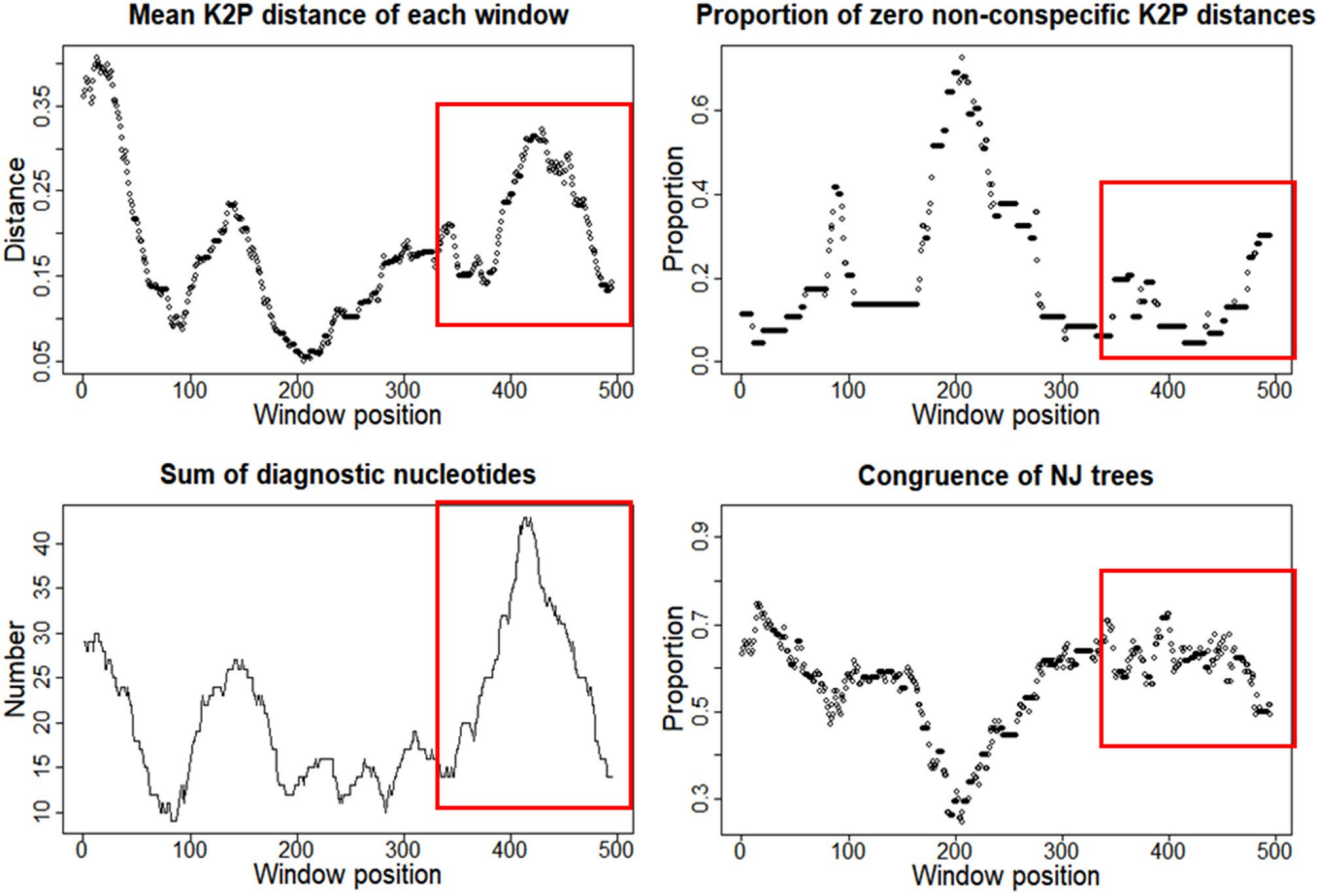
Screening for the best target region for mini-barcode across the 12S full alignment (565 bp) of 67 species using the sliding window analyses (SWAN) from SPIDER package. We analyzed (1) high mean K2P distance; (2) few zero pairwise non-conspecific distances; (3) high proportion of clades shared between the Neighbor-joining tree from the 12S full-length barcode and the tree constructed using only data from selected windows; and (4) high sum of diagnostic nucleotides. Rectangles represent the ideal region for primer design based on best indices of all criteria.

We aimed for conserved primer sites from the 12S full-length library (565 bp) and designed three primer sets with amplicons ranging from 171 to 193 bp, namely NeoFish_1, NeoFish_2, and NeoFish_3 (Table 1). All three primer sets recovered by Primer3 software targeted a similar amplicon region, differing by few base-pairs. The amplicon region started at position 639 and ends at the position 831 of the12S rRNA region of *Prochilodus costatus* (GenBank accession number NC_027690) (Fig. 1a). Primer Map showed that our target amplicon region does not overlap with other sets previously designed for the 12S region (i.e. Teleo1 and MiFishU); however, the primer NeoFish_3R uses almost the same annealing site as Teleo1-F (Fig. 1a). According to SWAN analyses conducted in SPIDER, the region that recovered the best indices of all criteria to design primers is within the 320 to 500 bp of the 12S full-length database, due to the higher sum of diagnostic nucleotides and congruence of NJ trees, as well as lower proportion of zero non-conspecifics (Fig. 2). The mean K2P distance of each window was highest at the beginning of our alignment, between 0 to 100 bp, but was also high within 320 to 400 bp range. Moreover, our chosen target region (~ from nucleotide 320 to 500 bp in Fig. 2) was surrounded by conserved regions with a low frequency of mismatches per primer, thus making it potentially useful to design group-specific primers in accordance with Primer3 choice of primer sites used by NeoFish_1, NeoFish_2, and NeoFish_3 (Fig. 1b).

Primer specificity analysis using Primer-BLAST showed that primer set NeoFish_3 had a lower amplification rate of non-targeted organisms such as bacteria, arthropods, mollusks, and mammalian species (including *Homo sapiens*) when compared to previously developed 12S primer sets (MiFishU and Teleo1) (Table 2). However, NeoFish_3 also showed cross amplification with birds, Lepidosauria, and amphibian sequences similar to previously published 12S markers (Table 2).

**Table 2:** Primer-BLAST results using the NeoFish_3 primer set and previously developed 12S markers (MiFishU—Miya et al., 2015; and Teleo1—Valentini et al., 2016). Numbers correspond to hits recovered for each taxonomic group.

Taxonomic groups	Primers sets		
	NeoFish_3	Teleo1	MiFishU
Bacteria	0	0	0
Arthropoda	0	33	16
Mollusca	0	62	15
Fish	> 1000	> 1000	> 1000
Amphibia	> 1000	> 1000	> 1000
Testudines	211	260	211
Crocodilia	57	50	1
Lepidosauria	> 1000	999	> 1000
Birds	> 1000	> 1000	> 1000
Mammalia	0	> 1000	> 1000
*Homo sapiens*	0	> 1000	865

### Species delimitation analyses of the full-length reference database

**Figure 3:**
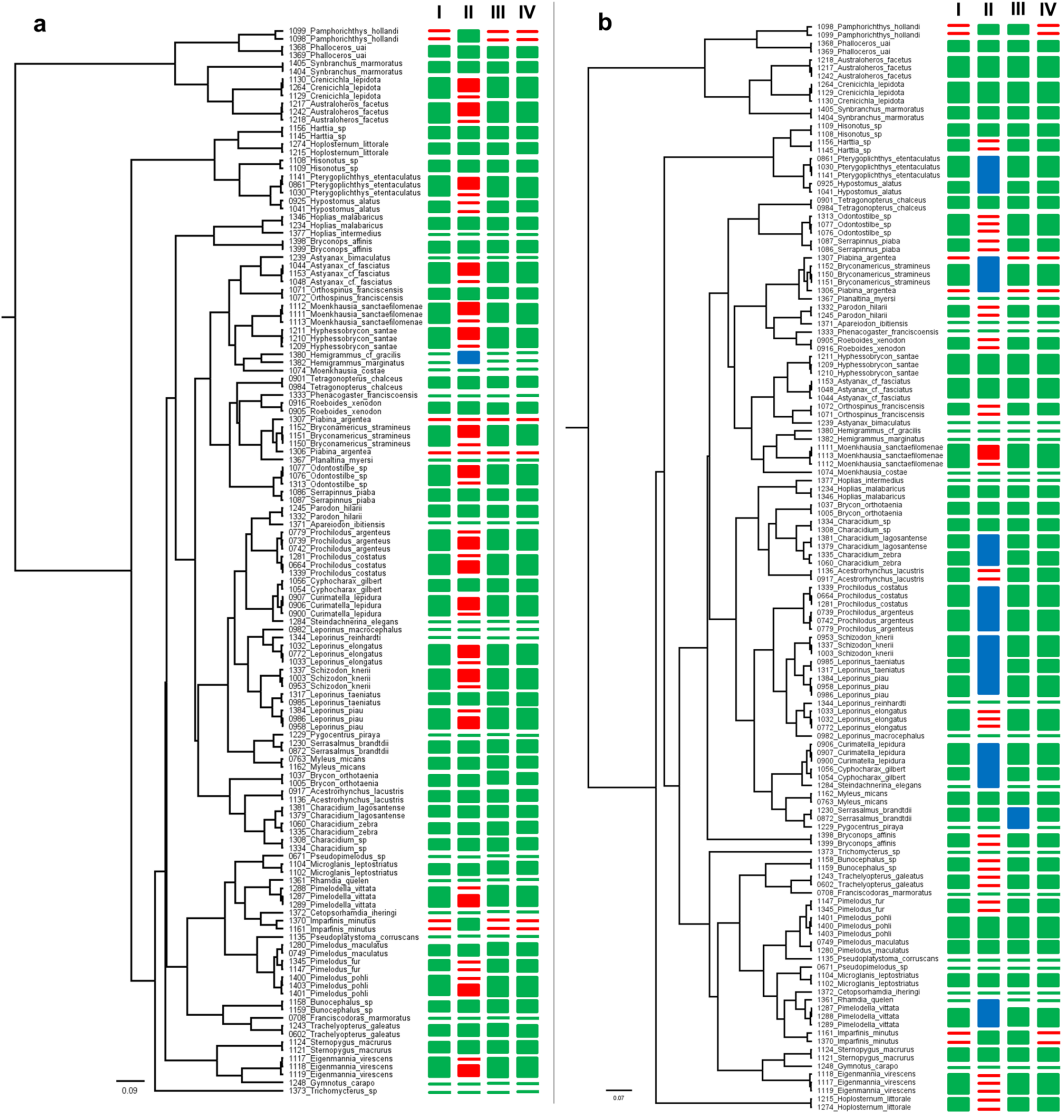
Bayesian phylogenetic ultrametric trees for 12S (**a**) full barcode and (**b**) mini-barcode for all species analyzed. MOTUs are represented by different sizes in accordance with each different species delimitation methods (I—GMYC, II—bPTP, III—ABGD, and IV—interspecific genetic distances thresholds). Green, blue and red colors represent MOTUs with a single species, multiple species, and different MOTUs for the same species, respectively.

Intraspecific genetic K2P divergences ranged from 0% to 2.06% (mean: 0.12%), 0% to 8.88% (mean: 0.92%) for intrageneric comparisons, and 0% to 9.97% (mean: 2.82%) for intrafamilial comparisons. Interspecific genetic distances ranged from 0.41% (*Prochilodus argenteus* vs *P. costatus*) to 32.33% (*Astyanax bimaculatus* vs *Synbranchus marmoratus*). The NJ dendrogram generated with all specimens showed species-specific branches (Supplementary Fig. S1).When considering species delimitation based on Bayesian methods, GMYC detected between 68 and 71 MOTUs (Fig. 3aI) and a threshold time of − 0.008, indicating the time before which all nodes reflect speciation events and after which all nodes reflect coalescent events. Maximum likelihood (ML) for the null model was 745.7335 and ML for GMYC model was 788.7312. The ML for the null model revealed the likelihood score of the model that considers that all the sequences belong to the same species, and the likelihood score of the model that splits the sequences into different species. In our case, it is highly significant (*P* = 0), indicating that there is more than one species in our sample. The bPTP revealed 86 MOTUs using a ML approach (Fig. 3aII), with branch support ranging from 0.487 to 1.

Species delimitation based on genetic distance using ABGD analysis detected between 57 and 70 MOTUs when varying the prior maximal distance from *P* = 0.021 to *P* = 0.001, respectively, using the simple distance (p-distance) (Fig. 3aIII). Four partitions, with prior maximal intraspecific distances ranging from 0.001 to 0.004 recovered 70 groups. Two partitions recovered 69 MOTUs, with prior maximal distances of 0.007 and 0.013. The ABGD partition of 70 groups (Fig. 3.aIII) of delimitated species was in agreement with the NJ clusters (Supplementary Fig. S1).

**Figure 4:**
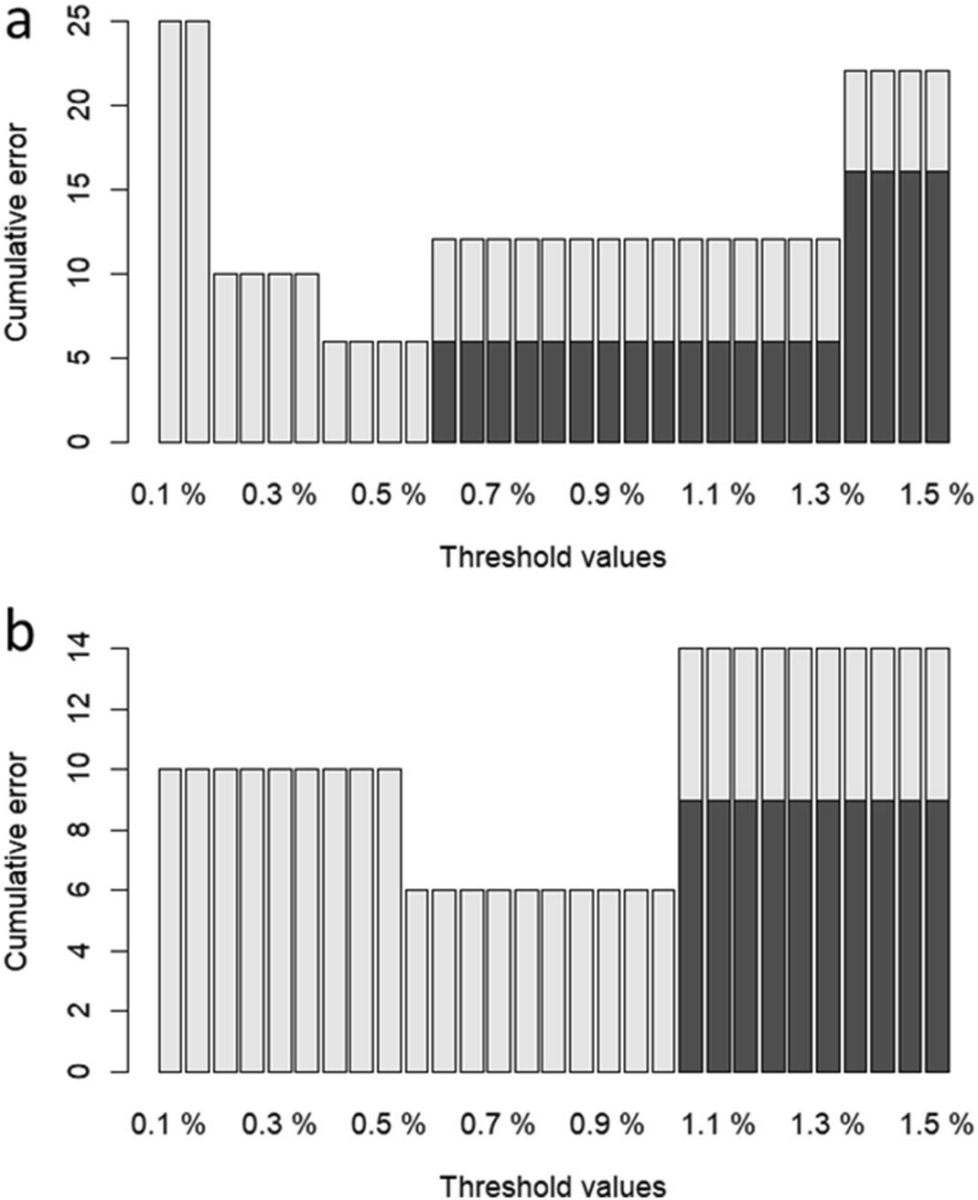
Threshold optimization for species delimitation for 12S (**a**) full length and (**b**) mini-barcode, showing the false positive (light grey) and false negative (dark grey) rate of fish species identification as pre-set thresholds change. Cumulative error is the sum of false positives + false negatives. For the full length, the percentages with lowest cumulative error (six) are between 0.4% and 0.55%. For the mini-barcode, the lowest cumulative error (also six) are within 0.55% and 1%.

The threshold analysis for species delimitation identified ranged from 0.4% up to 0.55% as the intraspecific values with the lowest number of cumulative errors (six). We used 0.4% as it is the most conservative percentage (Fig. 4a). Using the 0.4% threshold for species delimitation analysis (*threshID*), we recovered 70 MOTUs (Fig. 3aIV) within the 67 morpho-species previously identified by Carvalho et al. (2011). The overestimated three MOTUs are *Imparfinis minutus*, *Piabina argentea,* and *Pamphorichthys hollandi*.

### Species delimitation analyses of the mini-barcodes (193 bp)

The mini-barcode intraspecific genetic distance ranged from 0.0% to 1.14% (mean: 0.08%%), while the interspecific distances ranged from 1.14% to 49.03% (mean: 17.67%). Distance values for intrageneric ranged from 0.0% to 9.32% (mean: 1.03%) and intrafamilial ranged from 0.0% to 10.87% (mean: 3.37%). The NJ dendrogram generated with all specimens showed species-specific clades (Supplementary Fig. S1).

In silico species delimitation analyses based on Bayesian approaches, GMYC, and bPTP were able to recover all 67 species previously identified using traditional morphology-based identification. GMYC model recovered 70 genetic MOTUs (interval 68–71) (Fig. 3bI). The threshold time was − 0.005 and the ML for the null and GMYC model were 780.9562 and 821.8, respectively. The bPTP analysis revealed a total of 76 MOTUs (Fig. 3bII), with branch support ranging from 0.091 to 0.994.

ABGD was able to recover 59 to 67 groups when varying the prior maximal distance from *P* = 0.021 to *P* = 0.001. Five partitions, with prior maximal intraspecific distances ranging from 0.001 to 0.007, recovered 67 groups within our 12S mini-barcode database (Fig. 3bIII). The ABGD partition of 67 groups could delimitate most species in agreement with the NJ clusters (Supplementary Fig. S1); however, one group combined more than one species: (1) *Pygocentrus piraya* and *Serrasalmus brandtii* even though interspecific divergences could clearly differentiate this species (3.51%).

The intraspecific values with the lowest number of cumulative errors in the threshold analysis for species delimitation (six) were 0.55% up to 1%. We used 0.55%, which is the most conservative percentage in this case (Fig. 4b) and recovered 70 MOTUs (Fig. 3bIV) with this value. The overestimated three MOTUs (identified as “no ID” by *threshID* function) are *Imparfinis minutus*, *Piabina argentea,* and *Pamphorichthys hollandi*.

**Figure 5:**
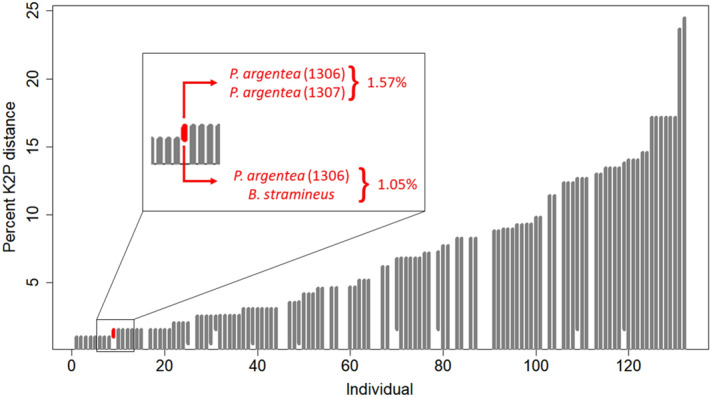
Barcode gap line plot for the 132 fish specimens. For each specimen in the dataset, the grey bars represent the gap between the highest intraspecific distance (bottom of the bar) and the lowest interspecific distance (top of the bar) representing the barcoding gap range. The red bar represents a specimen which these values overlap (intraspecific is higher than interspecific) meaning there is no barcoding gap.

Distance-based analyses performed using SPIDER showed similar results. In nearest-neighbor analysis, 99.2% of the sequences (112 out of 113—excluding 19 singletons) were correctly clustered, with only *P. argentea* (1306) being incorrectly clustered as nearest-neighbor of *Bryconammericus stramineus*. Barcoding gap analysis successfully recovered all species, since no overlap of intra and interspecific divergence was observed, except for *P. argentea* (1306) that has an intraspecific divergence of 1.57% with *P. argentea* (1307) and interspecific distance of 1.05% with *B. stramineus* specimens (Fig. 5).

## Discussion

We developed and curated a reference database for 67 fish species, belonging to 54 genera that are widespread across the Neotropical realm, and used it to develop a 12S mini-barcode marker and estimate a genetic distance threshold value for Neotropical fish species delimitation. Having a reference database associated with mini-barcode primer sets specific for Neotropical species is an important asset for DNA metabarcoding, especially when analyzing eDNA samples from such megadiverse fauna^21,22^.

The taxonomic resolution of 12S full and mini barcodes libraries provided enough molecular polymorphism to differentiate all 67 morpho-species. Moreover, the 12S full-length barcode (ca. 565 bp) was sufficient to discriminate all 70 MOTUs, which was in accordance with previous molecular (COI based) identifications of the same specimens^28^. Interestingly, the mini-barcode region’s (i.e. 193 bp—NeoFish_3) taxonomic resolution performed similarly to the full-length database, providing the same number of MOTUs when applying the GMYC and genetic distances thresholds analyses (70 MOTUs). The other analyses of the mini-barcode dataset overestimated the number of MOTUs (bPTP with 76) or underestimated it (ABGD with 67 MOTUs).

When performing genetic distance threshold analysis using the full-length library, we obtained a threshold value (0.40%, Fig. 4a) similar to our mini-barcode region (0.55%, Fig. 4b). Fish species delimitation threshold values based on the 12S region are an important reference for future studies using this marker, but they may need to establish a priori reference value when interpreting genetic distance data, such as the 2% widely used for COI^53^. Although we have analyzed several genera from all major Neotropical fish taxa, it is important to note that its value will be more robust and better reflect the real divergence between species when more species are added to our reference database.

Species delimitation and taxonomic resolution analyses revealed the potential of NeoFish_3 amplicons to reliably identify species, since there was no relevant disparity between full-length and mini barcode libraries for these analyses. Similar results were obtained for the COI gene, as a comparison between full-length and mini barcodes, especially when it was used in degraded samples. This demonstrates that the latter is informative for species-level sorting of: (1) major eukaryotic groups and archival specimens^45^; (2) moth and wasp museum specimens^54^, and; (3) several bird species^55^. However, few congeneric species have been analyzed in this study, and thus, to overcome this putative drawback, future analyses should include a higher number of species from the same genus to provide even more robust results.

SWAN analysis showed that the target NeoFish_3 amplicon would be the best region for taxonomic differentiation of species since it recovered the best indices in all established criteria (Fig. 2). However, we did not analyze the whole 12S gene of all species to proper compare the NeoFish_3 to other previously used amplicons (MifishU and Teleo1) using characteristics such as taxonomic resolution and best primer site. The target 12S rRNA gene region used to build our reference database represents approximately 60% of the 12S full-length gene (952 bp) (Fig. 1a) and includes only a small fragment of the 12S region amplified by the MiFishU marker and also the initial region of the forward Teleo1 (Fig. 1b).

In vitro tests showed that the newly developed NeoFish_3 marker is efficient and thus, was able to amplify the target region of the 12S rRNA gene from 22 tissue DNA extracts and environmental DNA recovered from an aquarium containing one fish species (Supplementary Table S1; Fig. S1). However, further evaluation of amplification success with samples obtained from Neotropical river basins using a DNA metabarcoding approach for a whole fish community is recommended, as different types of environmental samples will vary in patterns of DNA degradation and exposure to inhibitors^33^. Although 67 fish species represent a low percentage of the Neotropical freshwater fish species, they nevertheless account for the main Neotropical orders, since we include DNA of species from Characiformes, Cyprinodontiformes, Gymnotiformes, Perciformes, Siluriformes, and Synbranchiformes.

Amplification of non-target organisms has been previously reported as a drawback of universal eDNA available primer sets that led to the use of human blocking primers to avoid cross amplification. When comparing amplification of non-target taxa to previously designed primers sets (Teleo1 and MiFishU), a better specificity of NeoFish_3 was detected with our in silico PCR analysis. For Teleo1 and MiFishU the amplification rate for Mammalia, including *Homo sapiens*, was over 1000 sequences (Table 2), while the NeoFish_3 had no cross amplification of these. Moreover, when using the Teleo1 and MiFishU markers to assess fish communities diversity in French Guiana^21^ and Japan^31^, both papers report amplification of DNA from insects and mammals when analyzing eDNA samples. Such untargeted amplification and detection in eDNA studies may hamper the identification of rare species since it may consume most of the DNA sequences obtained^29,56^. However, before assuming that NeoFish_3 outperformed other 12S mini-barcode markers, in situ tests would be needed to check if there would indeed be lower amplification of non-targeted species.

Herein, we applied a powerful framework for the development and validation of a fish-specific primer set together with a custom reference database aimed at DNA metabarcoding analysis in the Neotropical realm. Species delimitation analyses strongly suggest that even when using a short region of the 12S mitochondrial region, we could discriminate each taxon to the species level. In addition, we were able to set an interspecific distance-based threshold for species delimitation that would be helpful throughout bioinformatics metabarcoding short reads analysis. Thus, our custom reference database and mini-barcodes markers are an important asset for an ecoregion scale DNA based biodiversity evaluation, such as eDNA metabarcoding, that can help with the complex task of conserving the megadiverse Neotropical ichthyofauna.

## Supplementary information

Supplementary Information.

## Data Availability

The newly generated sequences are available at GenBank under accession numbers MG755503 – MG755639.
