# Supplementary material for: New 12S metabarcoding primers for enhanced Neotropical freshwater fish biodiversity assessment
Source: Sci Rep. 2020 Oct 21;10:17966. doi: 10.1038/s41598-020-74902-3 (PMC7578065; doi:10.1038/s41598-020-74902-3)
Supplement: Supplementary file 1 — Supplementary Information. [file 41598_2020_74902_MOESM1_ESM.docx]

**New 12S metabarcoding primers for enhanced Neotropical freshwater fish biodiversity assessment**

David T. Milan^1^_;_ Izabela S. Mendes^1,2^; Júnio S. Damasceno^1^; Daniel F. Teixeira^1,2^; Naiara G. Sales^3,4^; Daniel C. Carvalho^1,2*^

*^*^Corresponding author: e-mail:* [*danielcarvalho@pucminas.br*](mailto:danielcarvalho@pucminas.br)

**SUPPLEMENTARY MATERIALS**

## Amplicons taxonomic resolution: Barcoding gap and nearest-neighbor

The barcoding gap analysis applied the highest intraspecific distance among its own species (*maxInDist()* function) and the lowest interspecific distance (*nonConDist()* function). Barcoding gap (Meyer and Paulay 2005) is a critical postulation in DNA barcoding as the amount of genetic variation within species and between species allows the differentiation of species. Moreover, we conducted ‘nearest-neighbor’ statistics using *nearNeighbour* function that finds the closest specimen to the target and returns as “true” if the closest specimen belongs to the same species and “false” if it does not, thus identifying instances where a risk of misidentification or ambiguity is likely.

##

**TableS1 – List of all species used to build the 12S reference database and the number of specimens for each species.**

| Species | Family | Order | No. of individuals |
| --- | --- | --- | --- |
| *Acestrorhynchus lacustris* | Acestrorhynchidae | Characiformes | 2 |
| *Apareiodon ibitiensis* | Parodontidae | Characiformes | 1 |
| *Astyanax bimaculatus* | Characidae | Characiformes | 1 |
| *Astyanax cf. fasciatus* | Characidae | Characiformes | 3 |
| *Australoheros facetus* | Cichlidae | Perciformes | 3 |
| *Brycon orthotaenia* | Bryconidae | Characiformes | 2 |
| *Bryconamericus stramineus* | Characidae | Characiformes | 3 |
| *Bryconops affinis* | Iguanodectidae | Characiformes | 2 |
| *Bunocephalus sp.* | Aspredinidae | Siluriformes | 2 |
| *Cetopsorhamdia iheringi* | Heptapteridae | Siluriformes | 1 |
| *Characidium lagosantense* | Crenuchidae | Characiformes | 2 |
| *Characidium sp.* | Crenuchidae | Characiformes | 2 |
| *Characidium zebra* | Crenuchidae | Characiformes | 2 |
| *Crenicichla lepidota* | Cichlidae | Perciformes | 3 |
| *Curimatella lepidura* | Curimatidae | Characiformes | 3 |
| *Cyphocharax gilbert* | Curimatidae | Characiformes | 2 |
| *Eigenmannia virescens* | Sternopygidae | Gymnotiformes | 3 |
| *Franciscodoras marmoratus* | Doradidae | Siluriformes | 1 |
| *Gymnotus carapo* | Gymnotidae | Gymnotiformes | 1 |
| *Harttia sp.* | Loricariidae | Siluriformes | 2 |
| *Hemigrammus cf. gracilis* | Characidae | Characiformes | 1 |
| *Hemigrammus marginatus* | Characidae | Characiformes | 1 |
| *Hisonotus sp.* | Loricariidae | Siluriformes | 2 |
| *Hoplias intermedius* | Erythrinidae | Characiformes | 1 |
| *Hoplias malabaricus* | Erythrinidae | Characiformes | 2 |
| *Hoplosternum littorale* | Callichthyidae | Siluriformes | 2 |
| *Hyphessobrycon santae* | Characidae | Characiformes | 3 |
| *Hypostomus alatus* | Loricariidae | Siluriformes | 2 |
| *Imparfinis minutus* | Heptapteridae | Siluriformes | 2 |
| *Leporinus elongatus* | Anostomidae | Characiformes | 3 |
| *Leporinus macrocephalus* | Anostomidae | Characiformes | 1 |
| *Leporinus piau* | Anostomidae | Characiformes | 3 |
| *Leporinus reinhardti* | Anostomidae | Characiformes | 1 |
| *Leporinus taeniatus* | Anostomidae | Characiformes | 2 |
| *Microglanis leptostriatus* | Pseudopimelodidae | Siluriformes | 2 |
| *Moenkhausia costae* | Characidae | Characiformes | 1 |
| *Moenkhausia sanctaefilomenae* | Characidae | Characiformes | 3 |
| *Myleus micans* | Serrasalmidae | Characiformes | 2 |
| *Odontostilbe sp.* | Characidae | Characiformes | 3 |
| *Orthospinus franciscensis* | Characidae | Characiformes | 2 |
| *Pamphorichthys hollandi* | Poeciliidae | Cyprinodontiformes | 2 |
| *Parodon hilarii* | Parodontidae | Characiformes | 2 |
| *Phalloceros uai* | Poeciliidae | Cyprinodontiformes | 2 |
| *Phenacogaster franciscoensis* | Characidae | Characiformes | 1 |
| *Piabina argentea* | Characidae | Characiformes | 2 |
| *Pimelodella vittata* | Heptapteridae | Siluriformes | 3 |
| *Pimelodus fur* | Pimelodidae | Siluriformes | 2 |
| *Pimelodus maculatus* | Pimelodidae | Siluriformes | 2 |
| *Pimelodus pohli* | Pimelodidae | Siluriformes | 3 |
| *Planaltina myersi* | Characidae | Characiformes | 1 |
| *Prochilodus argenteus* | Prochilodontidae | Characiformes | 3 |
| *Prochilodus costatus* | Prochilodontidae | Characiformes | 3 |
| *Pseudopimelodus sp.* | Pseudopimelodidae | Siluriformes | 1 |
| *Pseudoplatystoma corruscans* | Pimelodidae | Siluriformes | 1 |
| *Pterygoplichthys etentaculatus* | Loricariidae | Siluriformes | 3 |
| *Pygocentrus piraya* | Serrasalmidae | Characiformes | 1 |
| *Rhamdia quelen* | Heptapteridae | Siluriformes | 1 |
| *Roeboides xenodon* | Characidae | Characiformes | 2 |
| *Schizodon knerii* | Anostomidae | Characiformes | 3 |
| *Serrapinnus piaba* | Characidae | Characiformes | 2 |
| *Serrasalmus brandtii* | Serrasalmidae | Characiformes | 2 |
| *Steindachnerina elegans* | Curimatidae | Characiformes | 1 |
| *Sternopygus macrurus* | Sternopygidae | Gymnotiformes | 2 |
| *Synbranchus marmoratus* | Synbranchidae | Synbranchiformes | 2 |
| *Tetragonopterus chalceus* | Characidae | Characiformes | 2 |
| *Trachelyopterus galeatus* | Auchenipteridae | Siluriformes | 2 |
| *Trichomycterus sp.* | Trichomycteridae | Siluriformes | 1 |

**FigS1** Neighbor-Joining tree built with 132 samples of the 12S region (a) full length (565 bp) and (b) mini-barcode (193 bp) from 67 species.

**
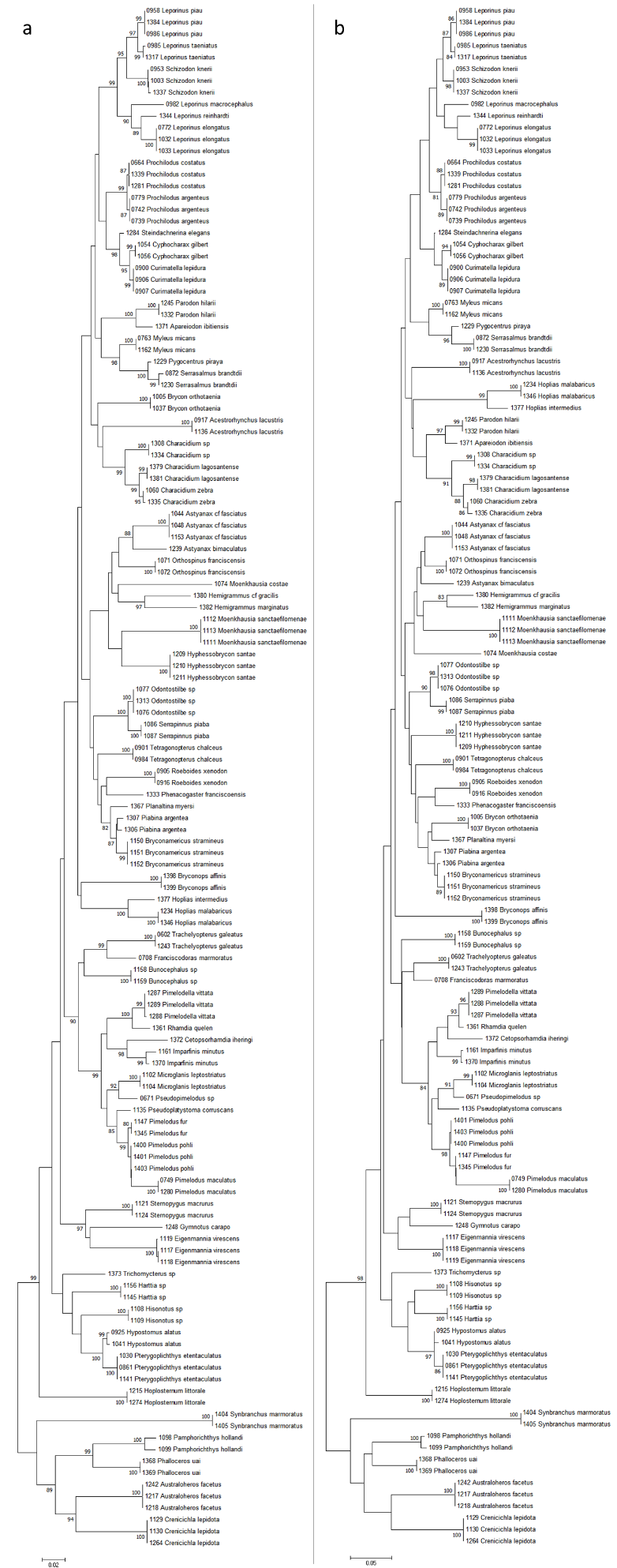
**

## *In vitro* tests: evaluation of primer efficiency

An aquarium (water volume 80 liters) containing one cichlid fish species (*Geophagus brasiliensis*) was prepared in a climate-controlled, aerated room receiving natural light. All experimental procedures were approved by the Animal Research Committee of Pontifical Catholic University of Minas Gerais (CEU PUC Minas permit number 021/2017) and followed the Brazilian guidelines for animal experimentation (MCTIC 2016).

We collected 500 mL of water using autoclaved plastic containers and filtered it immediately using a 0.45 µm membrane filter (cellulose acetate, Sartorius Stedim Biotech) and a vacuum filtration system (NALGENE Filter Holder). Filters were stored in sterile microcentrifuge tubes containing silica beads (Bakker et al. 2017) until DNA extraction. We extracted eDNA following the Salting Out extraction protocol adapted from Aljanabi & Martinez (1997), the same procedure we used for tissue samples when developing our reference database. We performed eDNA purification using the NucleoSpin Blood Purification Column (Macherey-Nagel, Duren, Germany). This two-phase eDNA purification was done to retrieve any PCR inhibitor that may have been carried over from the saline DNA extraction method.

To control for contaminants that may occur during water filtration, we used a chloride-bleach solution (50%) to wash all recipients, which were later rinsed with distilled water and ethanol. Negative controls consisted of filtering distilled water before conducting the filtration of the aquarium water. Filters of distilled water were submitted to DNA extraction and PCR.

PCR conditions for eDNA were the same as used for the *in vitro* mini-barcode primer sets using fish tissue amplification. As a positive control, we used a previously developed marker targeting any metazoan (mICOIintF-GGWACWGGWTGAACWGRWTAYCCYCC and jgHCO2198-TAIACYTCIGGRTGICCRAARAAYCA) (Günther et al. 2017) and genomic DNA extracted from tissue samples obtained from the cichlid specimens kept in the aquarium. PCR amplifications were sequenced bi-directionally using a BigDye Terminator v3.1 Cycle Sequencing Kit (Applied Biosystems), following the manufacturer's’ protocol on an ABI 3500 genetic analyzer. Molecular identification of recovered eDNA sequences was conducted using the BLAST tool in GenBank database (http://www.ncbi.nlm.nih.gov/blast) and by comparing it with the 12S DNA sequence recovered from the cichlid specimen.

The NeoFish_2 and NeoFish_3 performed similarly during *in vitro* tests, amplifying all species from all main Neotropical orders (Table S2). The mini-barcode primer set NeoFish_3 was chosen for further analyses since it targets an amplicon slightly larger (193bp) than NeoFish_2 (184bp). Environmental DNA of the pearl cichlid (*G. brasiliensis*) was successfully detected in the aquarium water samples using NeoFish_3 primer set and also the universal metazoan primer set (Günther et al. 2017), used as a PCR positive control. The recovered eDNA sequence had a consensus length of 196 bp (after trimming ambiguous ends) and 100% similarity with the *G. brasiliensis* specimen kept in the aquarium (Fig.S2). When searching for the eDNA sequence recovered on Genbank using BLAST, the highest match was observed with two *Geophagus* *brasiliensis* records, the most similar of them being the 12S region of the *Geophagus brasiliensis* mitochondrion (KU531434) with 98.76% similarity. Negative controls from water filtration, DNA extraction and PCR evidenced no contamination, since no positive amplification was observed on agarose gels (Fig.S2).

**FigS2.**  Environmental DNA amplification, sequence alignment, and Sanger DNA sequencing chromatogram of a sample obtained from an aquarium containing multiple specimens of the cichlid *G. brasiliensis*. Positive eDNA amplifications obtained using NeoFish_3 primer set with eDNA dilutions 1, 1:10, and 1:20 are depicted in the agarose electrophoresis gel. The recovered eDNA sequence was compared with a reference sequence from Genbank (KU531434.1) and with the 12S sequence obtained from the cichlid tissue (*G_brasiliensis*)


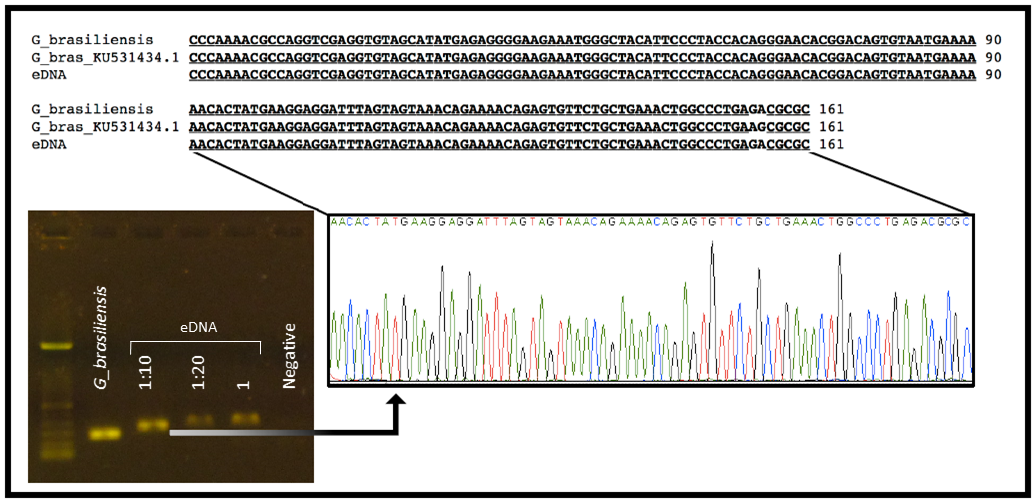


**TableS2.** Species used with each mini-barcode primer set for *in vitro* validation.

| **Species** | **Mini-barcode primer sets** | | | |
| --- | --- | --- | --- | --- |
|  | NeoFish_1 | NeoFish_2 | NeoFish_3 |  |
| *Characidium lagosantense* | X | X | X |  |
| *Crenicichla lepidota* |  | X | X |  |
| *Geophagus brasiliensis* | X | X | X |  |
| *Gymnotus carapo* |  | X | X |  |
| *Hisonotus sp.* | X | X | X |  |
| *Leporinus taeniatus* | X | X | X |  |
| *Parodon hilarii* | X | X | X |  |
| *Phalloceros uai* | X | X | X |  |
| *Pimelodus fur* | X | X | X |  |
| *Pimelodus maculatus* | X | X | X |  |
| *Pimelodus pohli* | X | X | X |  |
| *Prochilodus costatus* | X | X | X |  |
| *Pseudoplatystoma corruscans* | X | X | X |  |
| *Roeboides xenodon* |  | X | X |  |
| *Sternopygus macrurus* | X | X | X |  |
| *Tetragonopterus chalceus* |  | X | X |  |
